# Efficacy and safety of robotic radical hysterectomy in cervical cancer compared with laparoscopic radical hysterectomy: a meta-analysis

**DOI:** 10.3389/fonc.2024.1303165

**Published:** 2024-05-15

**Authors:** Zhen Dai, Fuqiang Qin, Yuxing Yang, Weiming Liang, Xiao Wang

**Affiliations:** The First Affiliated Hospital of Guangxi University of Science and Technology, Guangxi University of Science and Technology, Liuzhou, Guangxi, China

**Keywords:** cervical cancer, robotic, radical hysterectomy, laparoscopic, meta-analysis

## Abstract

**Introduction:**

Robotic radical hysterectomy (RRH) is a newly developed minimally invasive surgery that has been suggested as a substitute for laparoscopic radical hysterectomy (LRH). This meta-analysis aims to assess the clinical efficacy and safety of robot-assisted radical hysterectomy (RRH) for cervical cancer.

**Materials and methods:**

A systematic search was conducted in four databases (Medline, Embase, Web of Science, and CENTRAL) for studies comparing the utilization of RRH and LRH in the treatment of cervical cancer. The search included articles published from the inception of the databases up until July 18, 2023. Meta-analyses were conducted to assess several surgical outcomes, including operation time, estimated blood loss, length of hospital stay, pelvic lymph nodes, positive surgical margin, total complications, one-year recurrence rate, one-year mortality, and one-year disease-free survival rate.

**Results:**

Six studies were included for meta-analysis. In total, 234 patients were in the RRH group and 174 patients were in the LRH group. RRH had significantly longer operative time (MD=14.23,95% CI:5.27~23.20, P=0.002),shorter hospital stay (MD= -1.10,95% CI:-1.43~0.76, P <0.00001),more dissected pelvic lymph nodes(MD=0.89,95%CI:0.18~1.60, P =0.01) and less blood loss(WMD = -27.78,95%CI:-58.69 ~ -3.14, P=0.08, I^2 =^ 80%) compared with LRH. No significant difference was observed between two groups regarding positive surgical margin (OR = 0.59, 95% CI 0.18~2.76, P=0.61), over complications (OR = 0.77, 95% CI, 0.46-1.28, P=0.31), one-year recurrence rate (OR = 0.19, 95% CI 0.03-1.15, P=0.13), one-year mortality rate (OR = 0.19, 95% CI 0.03-1.15, P=0.07) and disease-free survival at one year (OR = 1.92, 95% CI 0.32-11.50, P=0.48).

**Conclusion:**

RRH is an increasingly popular surgical method known for its high level of security and efficiency. It has many benefits in comparison to LRH, such as decreased blood loss, a higher quantity of dissected pelvic lymph nodes, and a shorter duration of hospitalization. Further multicenter, randomized controlled trials with extended follow-up durations are necessary to conclusively determine the safety and efficacy of RRH, as no significant differences were observed in terms of positive surgical margin, postoperative complications, 1-year recurrence, 1-year mortality, and 1-year disease-free survival.

**Systematic Review Registration:**

PROSPERO, identifier CRD42023446653

## 1 Introduction

Cervical cancer is the predominant form of gynecological malignancy, with the 7th highest occurrence rate and the 10th highest fatality rate among all cancers globally (1). It poses a persistent danger to women worldwide, comprising approximately 8% of all occurrences of female cancer and overall cancer-related fatalities. Gynecologic oncologists have been seeking a surgical procedure that will minimize patient harm and decrease the rates of recurrence and mortality. Cervical cancer surgery has transitioned from open abdominal surgery to minimally invasive surgery.

Radical hysterectomy (RH) combined with pelvic lymphadenectomy has traditionally been a widely used treatment option for cervical cancer (2). While classical laparotomy has gained recognition, its use of a large incision and potential complications might result in discomfort and a less favorable prognosis (3). Since its initial report in the 1990s, it has been demonstrated that LRH is both more efficient and less risky compared to traditional laparotomy (4). The clearance of the Da Vinci surgical system by the US Food and Drug Administration has made robotic radical hysterectomy (RRH) a viable option for gynecologic surgery, offering a less intrusive method. The Da Vinci robotic surgical system enhances surgical precision and addresses the ergonomic limitations of LRH by providing surgeons with a more distinct and three-dimensional operative visualization (5). Nevertheless, it is important to acknowledge that there are several constraints associated with it, including the absence of perceptual feedback and the high expense involved (6). It is worthwhile to investigate if RRH offers more benefits compared to LRH in the treatment of cervical cancer.

The objective of this study was to comprehensively evaluate the available evidence in studies regarding the effectiveness and safety of RRH compared to LRA in treating patients with cervical cancer.

## 2 Materials and methods

### 2.1 Search strategy

This meta-analysis has been registered at PROSPERO with a registration number of CRD42023448639.The present study was conducted in accordance with the Preferred Reporting Project for Systematic Review and Meta-Analysis(PRISMA) 2020 guidelines. The following four literature databases were used for this study: Medline (1946 to Jul 18,2023), Embase (1974 to Jul 18,2023), Web of Science (1966 to Jul 18, 2023), and CENTRAL(1995 to Jul 18,2023).Searches for studies were completed by two independent investigators with the following searching strategy: “cervical tumor” and “robotic” and “laparoscopic” and (“randomized controlled trial” or “prospective study”). The search records of the four databases are detailed in **Supplementary Tables 1**–**4**. Manual search of relevant referable literature including related reviews and meta-analysis was preformed to identify other available studies.

### 2.2 Inclusion and exclusion criteria

Inclusion criteria were as follows:(1)patients were diagnosed as cervical cancer by the International Obstetrics and Gynecology Federation (FIGO), with stages not exceed IIB;(2) patients in the intervention group received RRH; (3)patients in the controlled group received LRH; (4)At least one of the following outcomes was reported: perioperative complications, hospital stay, time of surgery, intraoperative bleeding, number of lymph nodes removed during surgery, postoperative hospital stay, early postoperative complications or mortality.(5)Study type was prospective study or randomized controlled trial(RCT).

Literatures meeting the following criteria were excluded:(1) Other types of articles, such as animal studies,guideline,case reports, publications, meta-analyses, letters, reviews, editorials,pharmacological intervention,and protocols; (2)patients were diagnosed as other cancer, or cervical cancer with stages exceed IIB;(3)studies not comparing RRH versus LRH; (4) articles not written in English; (5)study type was retrospective study; (6) data cannot be extracted; (7)duplicate patient cohort.

### 2.3 Data extraction

Two investigators separately completed the extraction of relevant data for the included articles and entered the data into a uniform standard spreadsheet. When a doubt or disagreement occurred in the process of extracting the data, a third author was consulted before making a decision. The two extracted data copies were checked by a third reviewer. The extracted data were as follows: first author, year of publication, country, study design, sample size (RRH and LRH group), mean age, tumor size, FIGO stage, operation time, estimated blood loss, conversion rate, number of dissected pelvic lymph nodes, positive surgical margin, hospital stay, duration of postoperative complications, postoperative complications, total complications, one-year mortality, one-year recurrence rate, one-year disease-free survival rate.

### 2.4 Risk of bias assessment

The Newcastle-Ottawa Scale (NOS) was used by two independent reviewers to assess the risk of bias in the included studies (7). NOS was divided into 4 items: “research subject selection” (4 points), 1 item: “comparability between groups” (2 points) and 3 items of “outcome measurement” (3 points), with the highest score of 9 points. Literature with ≥6 points was assessed as high quality. If there are differences in the assessment results, the controversial sections will be addressed through group discussion.

### 2.5 Data analysis and statistical methods

EndNote (Version 20; Clarification Analysis) was used to manage the selection of retrieved studies, including duplicate deletion studies. All study findings were analyzed using Review Manager 5.3 (Cochrane Collaboration, Oxford, UK). The odds ratio (OR) with a 95% confidence interval (CI) was used to compare the binary variables. Continuous variables were compared using a weighted mean difference (WMD) with a CI of 95%. Median and interquartile ranges of the continuous data were converted to mean and standard deviation. For all meta-analyses, Cochrane Q p-values and I^2^ statistics were used to test for heterogeneity. If the heterogeneity was low or moderate (I^2^ <50%), the pooled data were analyzed using a fixed effects model (FEM); if the heterogeneity was high (≥50%), the random effects model (REM) was used. Statistical heterogeneity was assessed using a standard chi-square test and was considered significant at P <0.05. Potential publication bias was assessed by visually inspecting the funnel plot.

## 3 Results

### 3.1 Literature search

**Figure 1:**
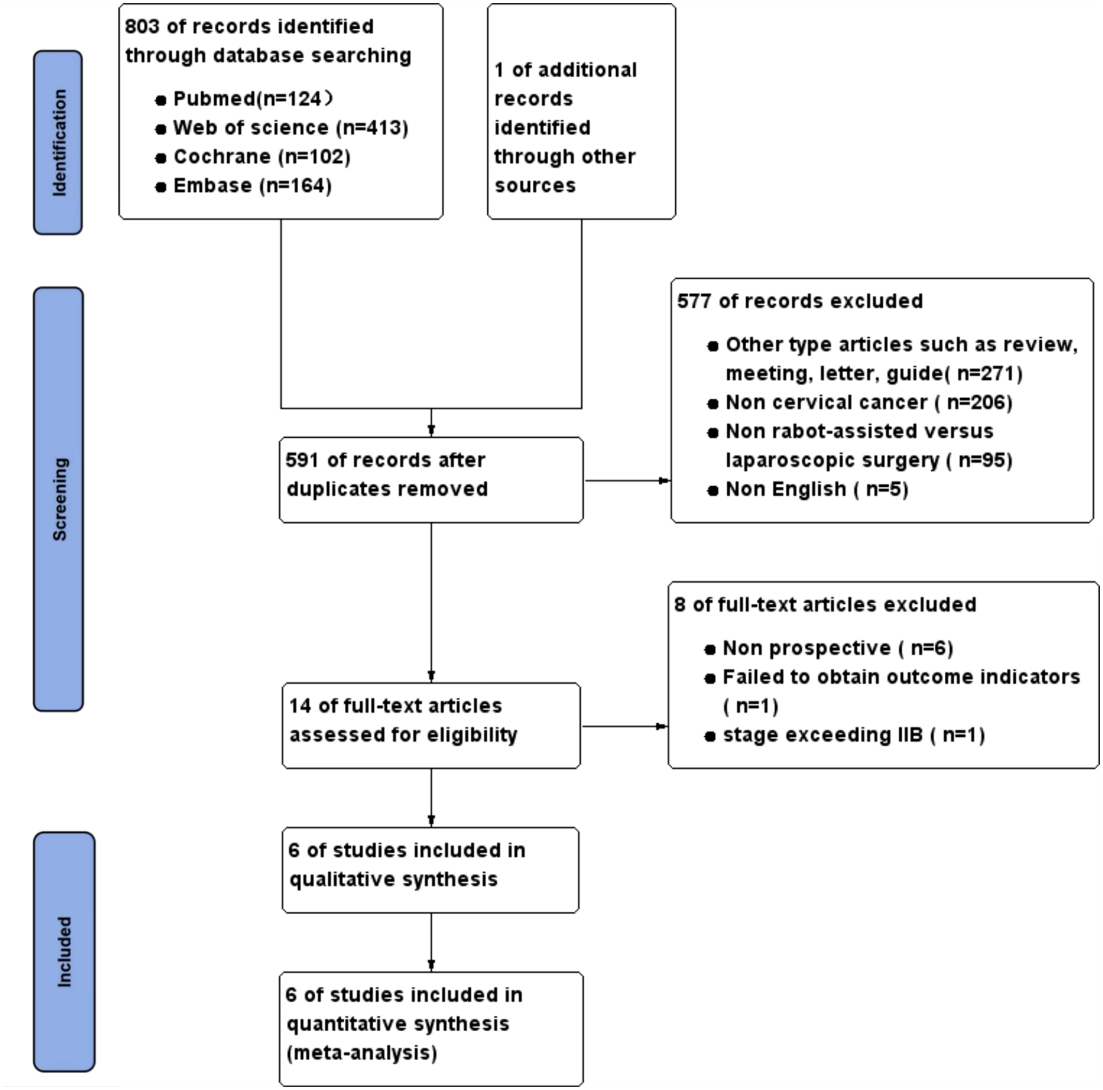
Flow chart of literature search strategies.

The precise procedure for selecting and excluding studies is illustrated in **Figure 1**. Following a methodical and thorough investigation, a grand total of 803 studies were obtained from 4 databases. One article was acquired by examining the citations of the recognized literature. After rigorously applying the inclusion and exclusion criteria, a total of six studies (8–13) were selected for the final meta-analysis.

### 3.2 Characteristics of the included studies

A total of six trials involving 408 patients were included in the analysis. All participants were female and over the age of 18. Of these, 234 patients were in the RRH group and 174 patients were in the LRH group. One of the research was a randomized controlled trial, whereas the other five studies were prospective non-randomized studies. **Table 1** displays detailed data and fundamental attributes of the patients enrolled in the six studies.

**Table 1:** Characteristics of the included studies.

First author/s, year	Study date	Study area	No. patients		Age, years (mea ± SD or median with range)		BMI (mea ± SD or median with range)		FIGO stage
			RRH	LRH	RRH	LRH	RRH	LRH	
**Nezhat,** **2008**	**2000-2006**	**USA**	**13**	**30**	**54.8** **(39-78)**	**46.8** **(29-63)**	**NA**	**NA**	**IA2 to IB2**
**Estape,** **2009**	**2006-2008**	**USA**	**32**	**17**	**55.0** **(33-78)**	**55.8** **(37-83)**	**29.7** **(24.8-36.3)**	**28.1** **(20.8-36.8)**	**IA2 to IIA**
**Vizza,** **2013**	**2010-2012**	**Italy**	**25**	**25**	**48.0** **(19-65)**	**49.0** **(26-75)**	**23** **(16-32)**	**22** **(18-30)**	**IB2 to IIB**
**Pellegrino,** **2017**	**2010-2016**	**Italy**	**34**	**18**	**46.9 ± 9.5**	**48.2 ± 13. 1**	**27,91** **± 5,75**	**23,93** **± 3,92**	**IA2 to IIA1**
**Luo,** **2018**	**2014-2015**	**China**	**30**	**30**	**47.1 ± 9.5**	**45.9 ± 8.9**	**NA**	**NA**	**IA to IIB**
**Ding,** **2019**	**2015-2016**	**China**	**100**	**54**	**65.0** **(62.0-67.0)**	**64.0** **(62.0-66.0)**	**22.5 ± 2.6**	**22.0 ± 2.4**	**IA2 to IIA2**

RRH, robotic radical hysterectomy; LRH, laparoscopic radical hysterectomy; NA, not available.

### 3.3 Risk of bias

All the six studies were of high quality. The results of the risk of bias assessment are summarized in **Table 2**.

**Table 2:** Assessment of the quality of the studies based on the NOS.

Study	Selections				Comparability of casesand controls on the basis of the design or analysis	Outcomes			Score
	Is the case definition adequate?	Representativeness of the cases	Selection of Controls	Definition of Controls		Ascertainment of exposure	Same method Of ascertainment for cases and controls	Non-Response rate	
Nezhat, 2008	*	*	*	*	*			*	6
Ricardo, 2009	*	*	*	*	**	*		*	8
Vizza, 2013	*	*	*	*	**				6
Pellegrino, 2017	*	*	*	*	**	*	*	*	9
Luo, 2018	*	*	*	*	**	*		*	8
Ding, 2019	*	*	*	*	**	*			6

·'High'quality choices were scored with a'*'. A maximum of one 'star'for each hitem within the 'Selection' and 'Outcome' categories; maximum of two '*'for ‘Comparability' category.

### 3.4 Clinical outcomes

A summary of the meta-analysis of all clinical outcomes was present in **Table 3**.

**Table 3:** Results of the meta-analysis.

Outcomes	No. of studies	Sample size	Heterogeneity	Overall effect size	95% CI of overall effect	P Value
		RRH LRH	I^2^(%) P Value			
**Operation time (min)**	**5**	**204 144**	**0 0.41**	**WMD=14.23**	**5.27 ~23.20**	**0.002**
**Estimated blood loss (mL)**	**5**	**204 144**	**80 0.0005**	**WMD=-27.78**	**-58.69~3.14**	**0.08**
**pelvic lymph nodes**	**6**	**234 174**	**87 <0.00001**	**WMD=0.89**	**0.18~1.60**	**0.01**
**positive of surgical margins**	**5**	**134 120**	**0 0.59**	**OR=0.70**	**0.18~2.76**	**0.61**
**Overall complications**	**6**	**234 174**	**74 0.002**	**OR=0.77**	**0.46~1.28**	**0.31**
**Length of hospital stay (days)**	**6**	**234 174**	**78 0.0004**	**WMD=-1.10**	**-1.43~-0.76**	**<0.00001**
**One year recurrence rate**	**5**	**202 149**	**17 0.31**	**OR=0.43**	**0.14~1.27**	**0.13**
**One year mortality**	**4**	**109 95**	**5 0.31**	**OR=0.19**	**0.03~1.15**	**0.07**
**Disease-free survival at One year**	**3**	**79 63**	**0 0.35**	**OR=1.92**	**0.32~11.50**	**0.48**

#### 3.4.1 Operative time

**Figure 2:**
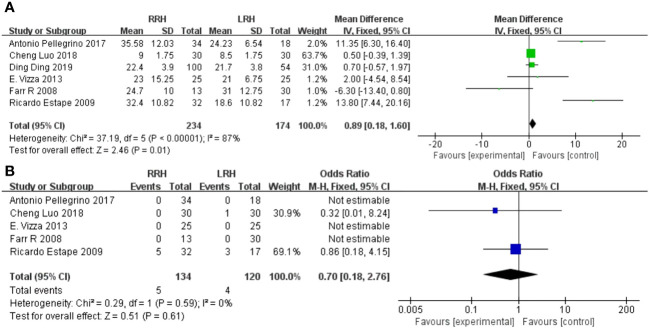
Forest plot of the meta-analysis for intraoperative parameters. **(A)** Operation time. **(B)** Estimated blood loss.

Operative time has been recorded in five trials. After analyzing the combined data from 5 investigations, it was determined that the RRH group had a significant lower operation time compared to the LRH group (WMD = 14.23,95% CI:5.27 ~ 23.20, P=0.002, I^2 =^ 0%) (**Figure 2A**).

#### 3.4.2 Estimated blood loss

Five studies provided data on estimated blood loss. The meta-analysis revealed that there was no statistically significant disparity in estimated blood loss between the two groups(WMD = -27.78,95%CI:-58.69 ~ -3.14, P=0.08, I^2 =^ 80%) (**Figure 2B**).

#### 3.4.3 Dissected pelvic lymph nodes

**Figure 3:**
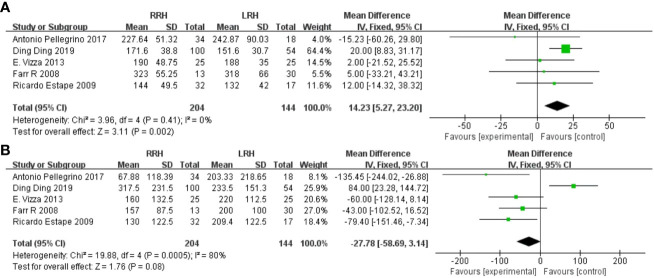
Forest plot of the meta-analysis for pathology details. **(A)** Number of pelvic lymph nodes. **(B)** Positive of Surgical margins.

The combined findings of six investigations demonstrated that the RRH group had a considerably higher number of dissected pelvic lymph nodes compared to the LRH groups(WMD = 0.89,95%CI 0.18 ~ 1.60, P =0.01, I^2 =^ 87%) (**Figure 3A**).

#### 3.4.4 Positive of surgical margins

A pooled analysis of five studies showed that there was no significant difference in positive of surgical margins between the RRH group and the LRH groups (OR = 0.70,95% CI 0.18 ~ 2.76, P=0.61,I^2 =^ 0) (**Figure 3B**).

#### 3.4.5 Length of stay

**Figure 4:**
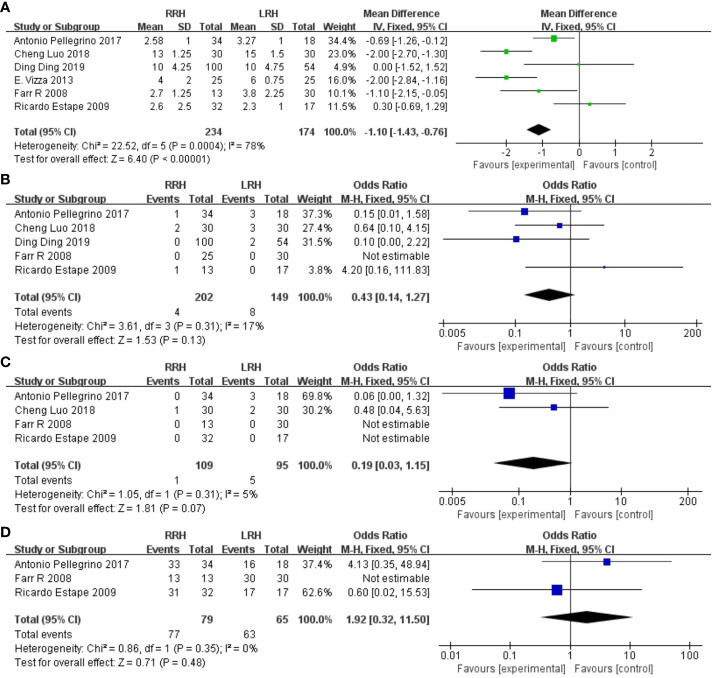
Forest plot of the meta-analysis for postoperative parameters. **(A)** Length of stay. **(B)** One-year recurrence rate. **(C)** One-year mortality. **(D)** Disease-free survival at one year.

Six trials provided data on the length of stay, and the combined analysis revealed that the length of stay was considerably shorter in the RRH group compared to the LRH group(WMD = -1.10,95% CI:1.43 ~ 0.76, P <0.00001, I^2 =^ 78%) (**Figure 4A**).

#### 3.4.6 One year recurrence rate

A pooled analysis of five studies showed no significant difference in one-year recurrence rates between RRH and LRH groups (OR = 0.43,95% CI:0.14 ~ 1.27, P=0.13, I2 = 17%) (**Figure 4B**).

#### 3.4.7 One-year mortality

A pooled analysis of four studies showed that there was no significant difference in one-year mortality between the two groups (OR = 0.19,95% CI:0.03 ~ 1.15, P=0.07) (**Figure 4C**).

#### 3.4.8 Disease-free survival at one year

A pooled analysis of three studies indicated that there was no significant difference in one-year disease-free survival between RRH and LRH groups (OR = 1.92,95% CI 0.32 ~ 11.50, P=0.48, I2 = 38%) (**Figure 4D**).

#### 3.4.9 Overall complications

**Figure 5:**
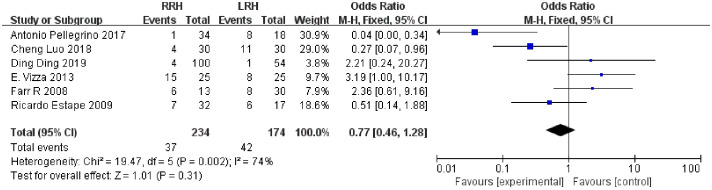
Forest plot of the overall complications.

Six studies suggested the total complications. The combined statistical results showed that there was no significant difference in the total postoperative complication rate between the two groups (OR = 0.77,95% CI,0.46 ~ 1.28, P=0.31, I2 = 74%) (**Figure 5**).

## 4 Discussion

Cervical cancer poses a persistent concern to women worldwide, comprising approximately 8% of all female cancer diagnoses and cancer-related fatalities (1).The development of cervical intraepithelial abnormalities and cancer can also be influenced by biological issues resulting from intricate molecular disruptions inside the vaginal area, in addition to the well-established causal impact of human papillomavirus (HPV) infection. Chronic oxidative stress occurs as a result of a decrease in oxygen levels in the vaginal area. Reactive oxygen species (ROS) and free radicals are currently unidentified causal agents that likely play a significant role in the development of cervical intraepithelial neoplasia (CIN) and cancer (14). Radical hysterectomy and lymph node dissection have emerged as crucial surgical techniques for the treatment of cervical cancer. Minimally invasive surgery offers notable benefits in terms of treatment efficacy, length of hospital stay, and recovery time compared to standard open surgery. Additionally, it has a more pronounced impact on lowering the physical and psychological distress experienced by patients (8). Over the past ten years, numerous studies have shown that robotic surgery is effective and safe for treating gynecological malignancies (15–17). There is an increasing level of excitement surrounding the use of minimally invasive techniques for treating cervical cancer. Recently, a minimally invasive treatment called RRH has been suggested as a substitute for LRH. The current study conducted a systematic review and meta-analysis to assess the safety and effectiveness of RRH compared to LRH in treating patients with cervical cancer.

Regarding the duration of the surgery, the meta-analysis results indicated that the RRH group had a shorter operation time compared to the LRH group. While certain prior investigations have shown comparable findings to ours (18, 19), other research have produced outcomes that contradict our own (20, 21). The possible explanation for this outcome could be attributed to the fact that the robotic system is a nascent technology, and various surgeons acquire proficiency in doing RRH and LRH in distinct ways. At the start of the learning process, the surgeon may suffer prolonged operation times when performing RRH for cervical cancer because to their limited expertise and knowledge in this area. Prior research has demonstrated that the duration of robotic surgery can be progressively reduced as the operating physicians gain greater skill and expertise (22).

Our findings indicated that the amount of blood lost during RRH was not significantly different from LRH, aligning with the results of several prior investigations (23). However, the findings of other studies diverged from our own results (19, 20, 23). The primary factor could be variations in the skill level and expertise of RRH between different operating physicians. There was a noticeable decrease in the need for blood transfusion during robot-assisted hysterectomy as doctors progressed from the initial stage to the advanced degree of proficiency (16). Prior research has indicated that the RRH group had a shorter duration of hospitalization compared to the LRH group. This can be attributed to the fact that RRH was linked to more comprehensive hemostasis and a reduced length of time spent in the hospital after surgery (14, 19, 20). The pooled results of this study are consistent with those previously reported.

In cases of gynecological malignancies, the spread of the disease often occurs through the lymph nodes. The accuracy of identifying lymph node metastases is crucial for determining the appropriate postoperative treatment and predicting the patient’s prognosis (24). Our findings suggest that RRH is superior than LRH in terms of the number of pelvic lymph nodes dissected. Prior research has also indicated that robotic surgery yields superior results in lymph node dissection for gynecological cancer compared to traditional laparoscopic surgery (25, 26). This is due to the improved clarity and balance of RRH, as well as the capability of its endoscopic operating device to replicate the movement of the human wrist. This allows for up to seven different angles to be reached beyond the hand. Surgeons have the ability to conduct a more comprehensive lymphadenectomy using a robotic device (25, 27). Therefore, the procedure in RRH is more accurate, enabling the removal of a greater number of lymph nodes.

This study demonstrated that there was no statistically significant disparity in outcomes between the two groups in relation to complications occurring during the observation period, recurrence of the condition within one year, mortality within one year, and disease-free survival within one year. In a similar vein, ChingHui et colleagues found no notable disparity in the overall rate of complications and disease-free survival between the RRH and LRH groups (19). Alberto A et al. also proposed similar five-year disease-free survival in the RRH and the LRH group (20).The possible explanation is that the effectiveness of the surgery is affected by the surgeon’s level of expertise, experience, and adherence to the device’s guidelines. The tumor prognosis for surgical treatment of pelvic tumors is substantially influenced by the technique and experience of the chief surgeon (28). There is no doubt that the number of surgical procedures will affect the surgical efficacy (29, 30). In the future, as robotic surgical systems continue to advance and surgical proficiency and coordination among RRH operators improve, there is potential for a reduction in mechanical damage and complications to patients, as well as a decrease in mortality and recurrence rates. Nevertheless, additional extensive RCTs with extended periods of observation are necessary to establish the effectiveness of RRH.

The scope of our study did not encompass the results of abdominal radical hysterectomy (ARH) and total laparoscopic radical hysterectomy(TLRH) for cervical cancer. Nevertheless, this is a highly significant matter. The publication of the Laparoscopic Approach to Cervical Cancer (LACC) trial in 2018 brought about a significant shift in the industry, revealing that minimally invasive surgery was linked to inferior oncological results when compared to an open approach (31).However, certain studies have shown divergent findings. A retrospective analysis found that death rate and recurrence rate were comparable between ARH and TLRH (p = 0.5514 and p = 0.1582, respectively) (32). Another study also indicated that introduction of a laparoscopic procedure in the surgical staging and treatment of cervical cancer patients did not have a detrimental effect on surgical or disease outcome, and this can be safely applied to the treatment of early stage cervical cancer (33).Furthermore, a retrospective study conducted across multiple institutions compared the outcomes of minimally invasive and open radical hysterectomy in patients with low-risk early-stage cervical cancer. The study seemed to provide further support to the growing evidence that laparoscopic radical hysterectomy does not lead to inferior 10-year outcomes compared to the open approach, specifically for low-risk patients (34). Corrado G et al. recently reported that minimally invasive approaches is not associated with different relapse patterns compared to ARH in FIGO stage IB1-IB2 cervical cancer, nor with a higher risk of distance metastasis and finally, without significant difference in term of DFS and OS (35). This evidence implies that the use of minimally invasive surgery for cervical cancer, which is thought to raise the risk of recurrence and have a negative impact on disease-free survival and overall survival in women, should be re-evaluated in future analyses to accurately assess the risks involved.

This study is the first meta-analysis to exclusively include prospective cohort studies or randomized controlled trials comparing RRH and LRH for treating cervical cancer. As a result, it has produced a strong and reliable conclusion regarding the safety and effectiveness of RRH. Nevertheless, our study does have several limitations. Initially, only a total of six papers were incorporated for this particular study. The statistical results of certain clinical outcomes may not accurately capture the disparities between the two groups due to the limited sample size. Furthermore, because to the limited duration of the studies included, we were unable to analyze the long-term oncology results between RRH and LRH, which is a crucial measure. Besides, the literature reviewed in this study did not provide any information regarding the surgeon’s skill level in RRH or LRH. This lack of information may contribute to the observed variability in the study. Hence, it is imperative to conduct further research in the future and increase the sample size in order to validate the findings of the current study.

In conclusion, our findings indicates that RRH is a secure and efficient surgical technique that is currently gaining prominence. It offers several advantages compared to LRH, including reduced blood loss, a greater number of dissected pelvic lymph nodes, and a shorter hospital stay. Given the importance of accurately identifying lymph node metastases in determining postoperative treatment and predicting patient prognosis, further research and application of RRH is highly justified. It is likely to result in improved long-term prognosis for cervical cancer patients. Additional multicenter, randomized controlled trials with longer follow-up periods are required to definitively establish the safety and effectiveness of RRH, as there were no notable disparities observed in terms of positive surgical margin, postoperative complications, 1-year recurrence, 1-year mortality, and 1-year disease-free survival.

## Data availability statement

The datasets presented in this study can be found in online repositories. The names of the repository/repositories and accession number(s) can be found in the article/**Supplementary Material**.

## Author contributions

ZD: Data curation, Formal analysis, Software, Writing – original draft. FQ: Conceptualization, Data curation, Formal analysis, Writing – original draft. YY: Resources, Software, Writing – original draft. WL: Methodology, Project administration, Writing – review & editing. XW: Funding acquisition, Investigation, Methodology, Writing – review & editing.
