# Supplementary material for: Efficacy and safety of robotic radical hysterectomy in cervical cancer compared with laparoscopic radical hysterectomy: a meta-analysis
Source: Front Oncol. 2024 May 15;14:1303165. doi: 10.3389/fonc.2024.1303165 (PMC11134290; doi:10.3389/fonc.2024.1303165)
Supplement: Supplementary file 1 [file Table_1.docx]

**Supplement Table 1.**The details of the searching record in Medline

| No. | Query | Results |
| --- | --- | --- |
| 1 | (((((((((((((((((((((("Uterine Cervical Neoplasms"[Mesh]) ) OR (Neoplasm, Uterine Cervical[Title/Abstract])) OR (Cervical Neoplasm, Uterine[Title/Abstract])) OR (Uterine Cervical Neoplasm[Title/Abstract])) OR (Neoplasms, Cervical[Title/Abstract])) OR (Cervical Neoplasms[Title/Abstract])) OR (Cervical Neoplasm[Title/Abstract])) OR (Neoplasms, Cervix[Title/Abstract])) OR (Cervix Neoplasm[Title/Abstract])) OR (Neoplasm, Cervix[Title/Abstract])) OR (Cervix Neoplasms[Title/Abstract])) OR (Cancer of the Uterine Cervix[Title/Abstract])) OR (Cancer of the Cervix[Title/Abstract])) OR (Cervical Cancer[Title/Abstract])) OR (Cancer, Cervical[Title/Abstract])) OR (Cervical Cancers[Title/Abstract])) OR (Uterine Cervical Cancer[Title/Abstract])) OR (Cancer, Uterine Cervical[Title/Abstract])) OR (Cervical Cancer, Uterine[Title/Abstract])) OR (Uterine Cervical Cancers[Title/Abstract])) OR (Cancer of Cervix[Title/Abstract])) OR (Cervix Cancer[Title/Abstract])) OR (Cancer, Cervix[Title/Abstract]) | 113,022 |
| 2 | (((Robotic[Title/Abstract]) OR (robotic[Title/Abstract])) OR (Robot[Title/Abstract])) OR (robot[Title/Abstract]) | 60,943 |
| 3 | ((((((Laparoscopes[Title/Abstract]) OR (Peritoneoscopes[Title/Abstract])) OR (peritoneoscopy[Title/Abstract])) OR (Celioscopes[Title/Abstract])) OR (celioscopy[Title/Abstract])) OR (Laparoscope[Title/Abstract])) OR (Laparoscopic[Title/Abstract]) | 131,497 |
| 4 | ((((((("Prospective Studies"[Mesh]) OR (Prospective Study[Title/Abstract])) OR (Studies, Prospective[Title/Abstract])) OR (Study, Prospective[Title/Abstract])) OR (Prospective Comparative Study[Title/Abstract])) OR (Prospective[Title/Abstract])) OR (prospective[Title/Abstract])) OR (prospectively[Title/Abstract]) | 1,052,975 |
| 5 | (randomized controlled trial [pt] OR controlled clinical trial [pt] OR randomized [tiab] OR placebo [tiab] OR clinical trials as topic [mesh:noexp] OR randomly [tiab] OR trial [ti]) NOT (animals [mh] NOT humans [mh]) | 1,426,929 |
| 6 | #4 OR #5 | 2,277,365 |
| 7 | #1 AND #2 AND #3 AND #6 | 124 |

**Supplement Table 2.**The details of the searching record in CENTRAL

| No. | Query | Results |
| --- | --- | --- |
| 1 | (Uterine Cervical Neoplasms OR Cervical Neoplasm, Uterine OR Neoplasm, Uterine Cervical OR Uterine Cervical Neoplasm OR Neoplasms, Cervical OR Cervical Neoplasms OR Cervical Neoplasm OR Neoplasms, Cervix OR Cervix Neoplasm OR Neoplasm, Cervix OR Cervix Neoplasms OR Cancer of the Uterine Cervix OR Cancer of the Cervix OR Cervical Cancer OR Cancer, Cervical OR Cervical Cancers OR Uterine Cervical Cancer OR Cancer, Uterine Cervical OR Cervical Cancer, Uterine OR Uterine OR Cervical Cancers OR Cancer of Cervix OR Cervix Cancer OR Cancer, Cervix):ti,ab,kw | 23,480 |
| 2 | (Laparoscopes OR Peritoneoscopes OR peritoneoscopy OR Celioscopes OR celioscopy OR Laparoscope OR Laparoscopic):ab,ti,kw | 23,482 |
| 3 | (Robotic OR robotic OR Robot OR robot):ab,ti,kw | 6,704 |
| 4 | (Prospective Studies OR Prospective Study OR Studies, Prospective OR Study, Prospective OR Prospective Comparative Study OR Prospective OR prospective OR prospectively):ab,ti,kw | 265672 |
| 5 | (randomized controlled trial OR randomized OR randomised OR randomization OR randomisa-tion OR rct OR randomly OR pla-cebo):ab,ti,kw | 1223024 |
| 6 | #4 OR #5 | 1271514 |
| 7 | #1 AND #2 AND #3 AND #6 | 102 |

**Supplement Table 3.**The details of the searching record in Embase

| No. | Query | Results |
| --- | --- | --- |
| 1 | 'uterine AND cervical AND 'neoplasms'/exp OR 'cervical neoplasm, uterine':ti,ab,kw OR 'neoplasm, uterine cervical':ti,ab,kw OR 'uterine cervical neoplasm':ti,ab,kw OR 'neoplasms, cervical':ti,ab,kw OR 'cervical neoplasms':ti,ab,kw OR 'cervical neoplasm':ti,ab,kw OR 'neoplasms, cervix':ti,ab,kw OR 'cervix neoplasm':ti,ab,kw OR 'neoplasm, cervix':ti,ab,kw OR 'cervix neoplasms':ti,ab,kw OR 'cancer of the uterine cervix':ti,ab,kw OR 'cancer of the cervix':ti,ab,kw OR 'cervical cancer':ti,ab,kw OR 'cancer, cervical':ti,ab,kw OR 'cervical cancers':ti,ab,kw OR 'uterine cervical cancer':ti,ab,kw OR 'cancer, uterine cervical':ti,ab,kw OR 'cervical cancer, uterine':ti,ab,kw OR 'uterine cervical cancers':ti,ab,kw OR 'cancer of cervix':ti,ab,kw OR 'cervix cancer':ti,ab,kw OR 'cancer, cervix':ti,ab,kw | 124,508 |
| 2 | ‘Robotic’/exp OR ‘robotic’:ab,ti,kw OR ‘Robot’:ab,ti,kw OR ‘robot’:ab,ti,kw | 97355 |
| 3 | ‘Laparoscopes’/exp OR ‘Peritoneoscopes’:ab,ti,kw OR ‘peritoneoscopy’:ab,ti,kw OR ‘Celioscopes’:ab,ti,kw OR ‘celioscopy’:ab,ti,kw OR ‘Laparoscope’:ab,ti,kw OR ‘Laparoscopic’:ab,ti,kw | 219,021 |
| 4 | ‘Prospective Studies’:ab,ti,kw OR ‘Prospective Study’:ab,ti,kw OR ‘Studies, Prospective’:ab,ti,kw OR ‘Study, Prospective’:ab,ti,kw OR ‘Prospective Comparative Study’:ab,ti,kw OR ‘Prospective’:ab,ti,kw OR ‘prospective’:ab,ti,kw OR ‘prospectively’:ab,ti,kw | 1,354,591 |
| 5 | 'randomized controlled trial'/exp OR 'randomized controlled trial':ti,ab,it OR 'randomized':ti,ab,it OR 'randomised':ti,ab,it OR 'randomization':ti,ab,it OR 'randomisa- tion':ti,ab,it OR rct:ti,ab,it OR 'randomly':ti,ab,it OR pla-cebo:ti,ab,it | 1,712,793 |
| 6 | #4 OR #5 | 2,840,860 |
| 7 | #1 AND #2 AND #3 AND #6 | 164 |

**Supplement Table 4.**The details of the searching record in Web of Science

| No. | Query | Results |
| --- | --- | --- |
| 1 | TS=(Uterine Cervical Neoplasms or Cervical Neoplasm, Uterine or Neoplasm, Uterine Cervical or Uterine Cervical Neoplasm or Neoplasms, Cervical or Cervical Neoplasms or Cervical Neoplasm or Neoplasms, Cervix or Cervix Neoplasm or Neoplasm, Cervix or Cervix Neoplasms or Cancer of the Uterine Cervix or Cancer of the Cervix or Cervical Cancer or Cancer, Cervical or Cervical Cancers or Uterine Cervical Cancer or Cancer, Uterine Cervical or Cervical Cancer, Uterine or Uterine or Cervical Cancers or Cancer of Cervix or Cervix Cancer or Cancer, Cervix or cervical cancer) | 438,047 |
| 2 | TS=(Laparoscopes OR Peritoneoscopes OR peritoneoscopy OR Celioscopes OR celioscopy OR Laparoscope OR Laparoscopic OR laparoscopy) | 216,416 |
| 3 | TS=(Robotic OR robotic OR Robot OR robot) |  |
| 4 | TS=(Prospective Studies OR Prospective Study OR Studies, Prospective OR Study, Prospective OR Prospective Comparative Study OR Prospective OR prospective OR prospectively) | 1,218,414 |
| 5 | TS=(randomized controlled trial OR controlled clinical trial OR randomized OR placebo OR clinical trials as topic OR randomly OR trial） | 3,083,364 |
| 6 | #4 OR #5 | 3,975,240 |
| 7 | #1 AND #2 AND #3 AND #6 | 413 |
